# Determination of Cocoa Flavanols and Procyanidins (by Degree of Polymerization DP1-7) in Cocoa-Based Products by Hydrophilic Interaction Chromatography Coupled With Fluorescence Detection: Collaborative Study

**DOI:** 10.1093/jaoacint/qsac007

**Published:** 2022-01-26

**Authors:** Ugo Bussy, Hong You, Catherine Kwik-Uribe

**Affiliations:** Mars, Incorporated, 6885 Elm St, McLean, VA 22101, USA; Eurofins Botanical Testing, US, Inc., 2951 Saturn St, Brea, CA 92821, USA; Eurofins Scientific, Inc., 2200 Rittenhouse St, Des Moines, IA 50321, USA; Mars, Incorporated, 6885 Elm St, McLean, VA 22101, USA

## Abstract

**Background:**

Cocoa flavanols and procyanidins (CF) are flavonoids whose consumption is associated with health benefits, resulting in increasing attention from consumers, industry, researchers, and regulators. Methods that can provide appropriate characterization and quantification of the distinct mixture found in cocoa-based products thus offer important scientific and commercial value.

**Objective:**

This study validated the precision of AOAC *Official Method of Analysis*^SM^**2020.05**, which measures CF with a degree of polymerization DP1-7.

**Method:**

Method precision (repeatability and reproducibility) was evaluated for seven cocoa matrixes in blind duplicates with total CF content from 1.0 to 500 mg/g. Ten of the 12 laboratories from multiple sectors invited to implement the method returned data for statistical analysis. Precision was evaluated per AOAC INTERNATIONAL guidelines for collaborative studies using RSD_r_ and RSD_R_ as indicators of method repeatability and reproducibility.

**Results:**

RSD_r_ ranged from 1.6 to 4.8%, and RSD_R_ ranged from 5.8 to 22.4%, demonstrating excellent within-laboratory repeatability and good method precision across different laboratories. RSD_R_ values were below 10% with the exception of chocolate, potentially due to very low CF content and sampling inhomogeneity.

**Conclusions:**

These data demonstrate that acceptable method repeatability and reproducibility is achieved when measuring cocoa flavanols and procyanidins using AOAC Method **2020.05** and support the advancement of the AOAC *Official Method of Analysis* status to Final Action for evaluated matrixes.

**Highlights:**

This collaborative study evaluated the repeatability and reproducibility of AOAC *Official Method of Analysis* **2020.05**.

Cocoa flavanols and procyanidins (CF) have been studied for more than two decades for cognitive and cardiovascular benefits potentially associated with their consumption (1–6). CF are also at the core of the largest clinical study ever organized on a botanical bioactive (COSMOS—COcoa Supplement and Multivitamin Outcomes Study; NCT02422745) (7) with outcomes expected to be publicly available in 2022. Flavanols from cocoa are made up of predominantly of (−)-epicatechin and, to a lesser extent, (+) and (−)-catechins (8, 9). In cocoa, these flavanol molecules also form oligomers, known as procyanidins, which are most often defined and measured by the degree of polymerization (DP) to accommodate for an increasing structural diversity as oligomers get larger (9). The sum of the monomeric flavanols (degree of polymerization of one or DP1) and procyanidins (DP ≥2) has been commonly defined as total cocoa flavanols (CFs).

In the past two decades, several methods have been proposed for the determination of flavanols and procyanidins in cocoa (10–14). The biggest hurdle faced by scientists in developing a quantitative and reliable methodology to measure CFs has been the lack of commercially available standards and reference materials to support instrument calibration that match natural procyanidin diversity found in cocoa. In this context, the National Institute of Standard and Technology (NIST) from the U.S. Department of Commerce recently produced a reference material (RM 8403) to support the development of new quantitative methodologies (15, 16). This material was incorporated as a calibrant in a hydrophilic interaction chromatography method to determine flavanols and procyanidins with different degrees of polymerization and received its First Action status of *Official Method of Analysis*^SM^ by AOAC INTERNATIONAL in 2020 (AOAC Method **2020.05**). This method was applied to a wide variety of cocoa-based products and validated in a single laboratory study (17). Preliminary data on method reproducibility have been published, but a full evaluation in a wider collaborative study remained necessary to guarantee method transferability, accessibility, and reproducibility.

This work summarizes the outcome of a collaborative study organized to evaluate the repeatability and reproducibility of AOAC Method **2020.05** (18). Seven matrices were selected with CF content ranging from 0.8 to 500 mg/g covering cocoa-based products such as chocolates, chocolate liquor, cocoa powder, cocoa extract, and dietary supplements. Results were processed following AOAC guidelines for collaborative study (19), estimating repeatability or RSD_r_ and reproducibility or RSD_R_ and compared to AOAC *Standard Method Performance Requirements* (SMPR^®^ 2012.001) for flavanol in food and beverages (20).

## Collaborative Study

*Collaborators.—*Twelve laboratories representing industrial, academic, commercial, and governmental institutions in four countries agreed to participate in this collaborative study. Invitation letters were first sent to provide clear instrumentation requirements to ensure the AOAC collaborative study requirements were met. After accepting participation, each laboratory received a package containing samples and consumables, while documents (method protocol, instruction to participant, and reporting form) were shared electronically. Study directors were available for remote technical support throughout the study.

*Study design.*—Following the instructions provided to collaborators, each laboratory was responsible for implementing the method and optimizing detector gain. The laboratory’s ability to run the method was first assessed by determining a blind practice sample (NIST baking chocolate RM 2384). The practice sample was subjected to the entire method protocol (including defatting), and results were reported alongside system suitability test results. After providing adequate results for the practice sample (% fat in 49–53% range and total CF in 7 to 9 mg/g range), each collaborator received authorization from study directors to move forward with the analysis of the remaining 14 samples (seven pairs of duplicates). These samples included seven matrixes (milk chocolate, NIST RM 2384 baking chocolate, cocoa powder, cocoa liquor, cocoa extract, ready-to-mix dietary supplement powder, and dietary supplement powder capsules) with CF content ranging from 1.0 to 500 mg/g. Each matrix was submitted in blind duplicates with their expected CF content hidden from participating labs. The exact matrix type had to be disclosed to collaborators as the method calls for matrix-specific sample preparation. The disclosure of matrix type was deemed low risk because of obvious and expected material differences between these common sample/product types. Each chocolate, cocoa powder, and liquor sample was defatted and analyzed in singlet and reported as such. A Microsoft Excel worksheet was provided to participants to record weights, retention times, and signal areas to support the assessment of system suitability and the determination of samples. This file included separate tabs to record data for the practice sample, collaborative study samples, and user feedback.

*Preparation of shipment.—*All samples were packaged on a single day. Chocolate and liquor solid samples were ground, homogenized, and aliquoted. Each sample contained approximately 10 g of samples if defatting was required and 1 g for samples that did not require defatting.

*Reference material.—*NIST cocoa extract reference material RM 8403 was provided (five sachets each containing 2 g) as the method calibrant (calibration reference standard). Another NIST cocoa-based reference material (RM 2384 baking chocolate) was included in the study as the practice and one of the study samples. NIST RM 2384 baking chocolate was defatted and analyzed three times by each laboratory (once as a practice sample and twice as a blind duplicate sample) with the objective to generate sufficient data to provide a benchmark for new implementation and routine performance monitoring of the method.

### Experimental

[Applicable for the determination of flavanol and procyanidin content (DP 1–7) of cocoa-based matrixes. The sum of monomeric (DP 1) and oligomeric fractions (DP 2–7) is reported as the total flavanol and procyanidin content.]

*Caution:* Solvents used are common-use solvents and reagents.

*Acetonitrile*.—Highly flammable, toxic, liquid irritant. Store in flammable liquid storage cabinet. Harmful if inhaled, swallowed, or absorbed through the skin. Use appropriate personal protective equipment and engineering controls, such as a laboratory coat, safety glasses, rubber gloves, and fume hood. Dispose of acetonitrile and solutions according to federal, state, and local regulations.

*Glacial acetic acid*.—Corrosive, flammable liquid. Store in an acid storage cabinet. Causes severe burns. Use appropriate personal protective equipment and engineering controls, such as a laboratory coat, safety glasses, face shield, heavy rubber gloves, and fume hood, when working with concentrated solutions. Dispose of acid and solutions according to federal, state, and local regulations.

*n-Hexane, methanol,* *and acetone.*—Flammable, toxic, liquid irritant. Store in a flammable liquid storage cabinet. Harmful if inhaled, swallowed, or absorbed through the skin. Use appropriate personal protective equipment and engineering controls, such as a laboratory coat, safety glasses, rubber gloves, and fume hood. Dispose of *n*-hexane and solutions according to federal, state, and local regulations.

### Principle

Chocolates, cocoa liquors, and cocoa powders are first extracted with hexane to remove their lipid components prior to extraction of flavanols and procyanidins. Flavanols and procyanidins (DP 1–7) are then extracted from these defatted materials and directly from cocoa extracts with an acidified aqueous acetone solvent system (acetone–water–acetic acid; AWAA). Finally, the extracts are cleaned up when necessary through solid-phase extraction or filtered and transferred to chromatography vials for HILIC HPLC analysis. This extraction procedure is highly effective and reproducible, and it does not result in loss or destruction of DP 1–7.

### Apparatus

- *HPLC system*.—Supporting back pressure of at least 400 bar, thermostated column compartment, solvent degasser, autosampler with temperature control and fluorescence detector: Waters ACQUITY H-Class (Waters Corp., Milford, MA), Agilent 1200/1260/1290 (Agilent Technologies, Santa Clara, CA), or similar.
- *Chromatography data acquisition software*.—Agilent ChemStation Plus Family, Revision C.01.09, Waters Empower 3 or equivalent.
- *HPLC column.*—Torus Diol 100 × 3.0 mm id, 130 Å, 1.7 µm particle size (Waters Corp.; Cat. No. 186007611), or equivalent.
- *Sonic bath*.—Capable of sonication and heating to at least 50°C (VWR, West Chester, PA; Model 150D), or equivalent.
- *Volumetric flasks*.—5, 10, 25, 50, or 100 mL.
- *Syringe filters*.—PTFE, 0.45 µm, 13 mm (Nalgene, Rochester, NY; Cat. No. 187-1345), or equivalent.
- *HPLC vials/caps*.—VWR (Cat. No. 608216-1232), or equivalent.
- *SPE cartridges*.—MCX PRiME, 30 µm, 150 mg/6 cc (Waters Corp.; Cat. No. 186008919).
- *Vacuum manifold*.—24 position (Phenomenex, Torrance, CA; Cat. No. AH0-6024), or equivalent.
- *Syringes*.—3 mL (VWR; Cat. No. BD309586), or equivalent.
- *Disposable centrifuge tubes*.—15 and 50 mL (VWR; Cat. Nos 21008-210 and 240), or equivalent.
- *Centrifuge*.—Capable of 1700 rcf. (Fisher Scientific, Hampton, NH; Cat. No. 75-009-261), or equivalent.
- *Vortex mixer*.—Fisher Scientific (Cat. No. 02-215-365), or equivalent.
- *Analytical balance*.—Readability to 0.1 mg.
- *Graduated cylinder*.—Fisher Scientific (Cat. No. 08552-4F), or equivalent.

### Reagents

- *Water*.—Millipore quality or 15MΩ·cm (EMD Millipore, Billerica, MA), or equivalent.
- *Hexanes*.—HPLC grade (Fisher Scientific; Cat. No. H303-4), or equivalent.
- Methanol.—HPLC grade (Fisher A454-4), or equivalent.
- *Acetone*.—HPLC grade (Fisher A929-4), or equivalent.
- *Acetonitrile*.—HPLC grade (Fisher A998-4), or equivalent.
- *Acetic acid*.—Glacial (Mallinckrodt Baker, Inc., Phillipsburg, NJ; Cat. No. 9534-33), or equivalent.
- *Mixture of calibration standard*.—Cocoa extract calibrant (NIST RM# 8403), or equivalent. Purity as indicated on the certificate of analysis.
- *Extraction solution AWAA*.—Combine 700 mL acetone, 300 mL purified water, and 10 mL glacial acetic acid (70 + 30 + 1). Solution mixture referred to as AWAA is used for calibration standards, as well as for extraction of flavanols and procyanidins from test samples.

### System Conditioning and Suitability

Fluorescence detector (FLD) performance varies from manufacturer to manufacturer and even within instruments from the same manufacturer. To quantitatively measure monomers (DP 1) through heptamers (DP 7) in a single measurement, the dynamic range must be optimized. Photomultiplier tube (PMT) gain setting for monomer (DP 1) needs to be established to ensure linearity of the signal, and yet still retain maximum sensitivity for hexamer (DP 7), typically present in much a lower quantity in cocoa-based materials. The instructions below guide this dynamic range optimization process.

**Figure 1.**
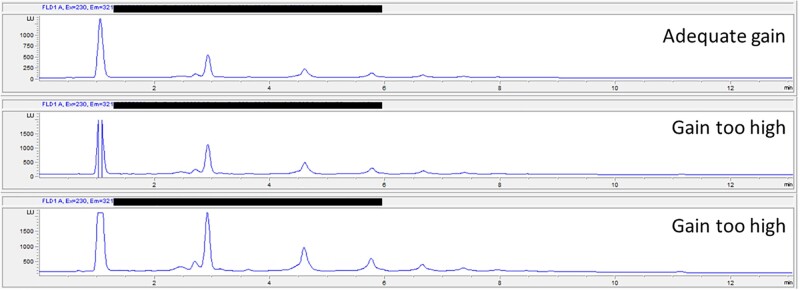
WS5 HPLC trace showing DP 1–7. Sample was run with an Agilent 1290 system at a PMT setting of 13, 14, and 15. Valley-to-valley integration is shown. The main point of this figure is to highlight the impact of gain selection on peak shape.

- *FLD sensitivity/dynamic range optimization and reproducibility*.—This step is critical to adequate implementation of the method and shall be performed every time the method is implemented on a new instrument or significant maintenance is performed on the instrument.
  1. Prepare a stock solution of cocoa extract calibrant in AWAA at 0.2 mg/mL by weighing accurately 20 mg cocoa extract calibrant into a 100 mL volumetric flask. Dilute to volume with AWAA solution. Prepare fresh. Do not store.
  2. Select an appropriate starting sensitivity level (i.e., gain setting) on the FLD of the HPLC system; often, one can begin at instrument default. Injection volume is 2 µL.
  3. Inject 0.2 mg/mL working solution of cocoa extract calibrant three times using the HPLC conditions specified in the *HPLC* *Parameters* section.
  4. Observe whether the DP1 peak is of normal shape and on scale (*see*Figure 1 for reference).
  5. If DP1 peak shape is normal and on scale, repeat steps **(a)***(4)* and *(5)* at the next most sensitive detector gain setting. Consider that you might have to reduce the gain setting (e.g., PMT from 14 to 13) to ensure the proper dynamic range and to be able to measure all procyanidins.
  6. Continue this process until the most sensitive detector gain setting for 0.2 mg/mL cocoa extract calibrant working solution has been identified.
  7. Once the optimum gain settings are identified, perform three subsequent injections of cocoa extract calibrant at 0.2 mg/mL. RSD, % on DP1 signal area must be ≤2%.
- *System suitability for analysis.—*This step is essential to verify proper instrument performances during each analysis. System suitability must be met for results to be valid.
  1. Each sequence must include 10 subsequent injections of check working standard at 0.1 mg/mL (preparation described in the *Extraction of Flavanols and Procyanidin*s section). The five first injections are not evaluated and are used only to ensure complete the equilibration of the column. The injections 6–10 are evaluated for system suitability.
    - The relative standard deviation on signal area for each degree of polymerization must meet the following acceptance criteria: RSD, % ≤2% for DP1, ≤2% for DP2, ≤5% for DP3, ≤5% for DP4, ≤10% for DP5, ≤15% for DP6, and ≤15% for DP7.
    - The average total CF determined on check working standard (injections 6–10) must be ≥90% and ≤110% of the expected value (referring to working standard certificate of analysis).
  2. Each sequence must include calibration curve levels 1–5 (preparation described in the sample preparation section). The coefficient of determination (r^2^) must be ≥0.99 for each DP1–7.
  3. Each sequence must include bracketing standard (check working standard) injection every 10 sample injections and must be followed by a blank injection (AWAA) prior to additional sample analysis.
    - System drift is verified against check working standard injections 6–10. Acceptable performances are recoveries in the check working standard of ±5, 10, and 20%, respectively, for DP1–4, DP5, and DP6–7.
    - Retention time for each DP in check working standard injection must be within 10% of the average retention time determined across check working standard injections 6–10.

### Preparation of Cocoa Extract Calibration Solutions

- *Stock solution of cocoa extract calibrant*.—Weigh 20 mg cocoa extract calibrant into a 100 mL volumetric flask and dilute to volume with AWAA. This will be the 0.2 mg/mL standard stock solution and will be used as the level 5 working standard.
- *Dilutions*.—Prepare the dilutions using the AWAA solvent, following the dilution scheme in Table 1 (e.g., dilute the stock solution of cocoa extract calibrant 2.0 mL into a 10 mL volumetric flask giving level 1 working standard at 0.04 mg/mL). Prepare fresh. Do not store. The concentration for each DP can be calculated at each level of the calibration curve following Equation 1.
  (1) $$C_{\mathrm{DPn}}\left(\text{mg}/\text{mL}\right)=\text{Stock}\;\text{concentration}\;\left(\text{mg}/\text{mL}\right)/df_{\text{calibrant}}\times \text{mass}\;\text{fraction}\;\text{DPn}\;\left(g/g\right)$$
  where Stock concentration is the concentration of the stock solution of cocoa extract calibrant (prepared as described in the *System Conditioning and Suitability* section); *df*_calibrant_ is the dilution factor used to create the calibration curve point (Table 1); and the mass fraction DPn is the declared mass fraction of the individual DPs found in the NIST documentation.
- *Check working standard*.—Weigh 20 mg cocoa extract calibrant into a 100 mL volumetric flask and dilute to volume with AWAA. Pipette 5 mL into a 10 mL volumetric flask and dilute to volume with AWAA. This will be the 0.1 mg/mL check working standard stock solution. Correct mass using purity of each DP1–7 following Equation 1.
- *Sequence table: blanks, standards, and test samples*.—Cocoa extract calibration solutions are run routinely prior to sample analysis. *See*Table 2 for a typical sequence that includes running system suitability samples (level 3), calibration solutions (levels 1–5), and a check sample (around level 3 concentration but a different preparation as described above).

**Table 1.** Preparation of cocoa extract calibration solutions

Level	Cocoa extract calibrant stock solution, mL	Total volume, mL	Concn, mg/mL	Dilution factor, *df*
1	2.0	10	0.04	5
2	4.0	10	0.08	2.5
3	6.0	10	0.12	1.67
4	8.0	10	0.16	1.25
5	N/A	100	0.20	1

**Table 2.** Sequence of samples for a batch of running test samples

No.	Sequence of sample types	Notes/comments
1	Blank	–
2	System suitability solutions ×10	Check working standard
3	Calibration solutions	Working standard levels 1–5
4	Blank	–
5	Test samples	10-Sample run (or practice samples)
6	One check sample	Check working standard
7	Blank	–

### Removal of Lipid Fraction

For sample types expected to contain more than 10% fat (Table 3), weigh approximately 5 g sample into a labeled 50 mL disposable centrifuge tube. Fill tube(s) to the 45 mL mark with hexane and cap tightly. Vortex at least 1 min to facilitate complete dispersion. Place tube(s) into a sonic bath at 50°C and sonicate for 5 min. Remove centrifuge tubes from the sonic bath and centrifuge all tubes for 5 min at 1700 rcf. Decant the hexane phase into a pre-tared beaker. Repeat this procedure twice more, combining the hexane layers, so that the extraction has been performed a total of three times. Allow the residual solids and hexane layer to dry in an appropriate fume hood until there is no evidence of remaining hexane. Weigh the residual solids from the hexane layers. Calculate the percent fat as the amount of residual solids from the hexane layer divided by the initial sample weight times 100%.

**Table 3.** Sample amount for extraction process

	Weight, mg	Volume, mL	Lipid removal with hexane wash	Sonication	Centrifuge	SPE	Dilution	Filtering	Dilution volume, DV, mL
Milk chocolate	2000	5	Yes	5 min at 50°C	5 min at 1700 rcf	MCX PRiME	2.5 in 25 mL	None	50
Baking dark chocolate	300	10	Yes	5 min at 50°C	5 min at 1700 rcf	MCX PRiME	2.5 in 25 mL	None	100
Cocoa liquor	260	10	Yes	5 min at 50°C	5 min at 1700 rcf	MCX PRiME	2.5 in 25 mL	None	100
Cocoa powder	500	10	Yes	5 min at 50°C	5 min at 1700 rcf	MCX PRiME	2.5 in 25 mL	None	100
Drink mix	120	25^a^	None	5 min at 50°C	5 min at 1700 rcf	None	1 in 10 mL	PTFE 0.45 µm	250
capsules	32	20	None	5 min at 50°C	5 min at 1700 rcf	MCX PRiME	2.5 in 25 mL	none	200
Cocoa extract	50	50 (flask)	None	none	none	None	1 in 10 mL	PTFE 0.45 µm	500

a Drink mix designed to be water soluble. To enhance extraction recovery, drink mix was first dissolve in 7.5 mL of HPLC water, sonicated, and then mixed with 17.5 mL of acetone–acetic acid (100:1.5).

### Extraction of Flavanols and Procyanidins

Accurately weigh the appropriate amount of the sample (defatted if appropriate as defined in *Removal of Lipid Fraction* section) into a 50 mL disposable centrifuge tube according to Table 3. Accurately add the appropriate amount of AWAA. Hand-shake briefly (until all solid is wetted with solvent) and vortex as needed, until all solid is wetted to facilitate dispersion. Place sample tubes into a 50°C sonic bath for 5 min. Centrifuge all tubes for 5 min at 1700 rcf.

Certain matrixes do not require SPE cleanup (e.g., cocoa extract) and can therefore be diluted to the desired concentration after dissolution according to Table 3. After dilution, filter using a 0.45 µm PTFE syringe filter and transfer to a HPLC autosampler vial.

Unless demonstrated to be unnecessary for the matrix, clean up extraction solution using a SPE MCX PRiME cartridge. Sample cleanup eliminates the accumulation of matrix components on the column, which leads to poor analytical performance. Perform conditioning of the SPE bed with 2 mL AWAA on a vacuum manifold. Do not allow the packing bed to dry at any time prior to loading the sample. After conditioning the column with AWAA, place a 15 mL centrifuge tube in the vacuum manifold to collect and load 2.5 mL supernatant (extraction) solution (nota bene: centrifuge tube will contain approximately 14.5 mL of solution; ensure that SPE outlet is not dipping in solution, causing overflow). Move extraction solution through the cartridge at low flow rate until 1–2 mm remain on top of the sorbent. Load 6 mL of AWAA and slowly move through the cartridge using vacuum; repeat this step once (total of 12 mL of AWAA). Remove the tube from the vacuum manifold, transfer contents to a 25 mL volumetric flask, and dilute with AWAA. Homogenize the flask and transfer approximately 1 mL to a HPLC autosampler vial (no filtering is required after SPE cleanup).

*Note:* Necessity for SPE cleanup and/or scale can be reconsidered pending demonstration that the elimination of the cleanup does not impact method performances and the ability to demonstrate system suitability.

### HPLC Parameters

- *Column and autosampler conditions*.—The column is a Torus diol (100 × 3.0 mm id, 1.7 µm, 130Å particle size). Hold the column temperature at 50°C. The flow rate is 1 mL/min, and typical injection volume is 2 µL. Set the autosampler to, and hold at, 5°C. Equilibration of the column with 50/50 solvent A/solvent B for at least 10 min prior to analysis may be needed.
- *Solvents and gradient*.—The mobile phase is a binary gradient (solvents A and B) consisting of (A) acetonitrile–acetic acid (98 + 2, v/v) and (B) methanol–water–acetic acid (95 + 3 + 2, v/v/v). The starting mobile phase condition is 0% B; hold isocratic for 0.37 min. Subsequently, ramp solvent B to 45% over 10.03 min and to 95% B 0.25 min thereafter. Hold at 95% B for 2.35 min prior to returning to starting conditions (0% B) over 0.10 min. Total run time is 13.10 min. Postrun equilibration is 3 min.
- *Fluorescence detection.—*Conduct fluorescence detection with an excitation wavelength of 230 nm and emission wavelength of 321 nm. Set the PMT to a level established per the *System* *Suitability* section prior to conducting analyses.

### Integration

**Figure 2.**
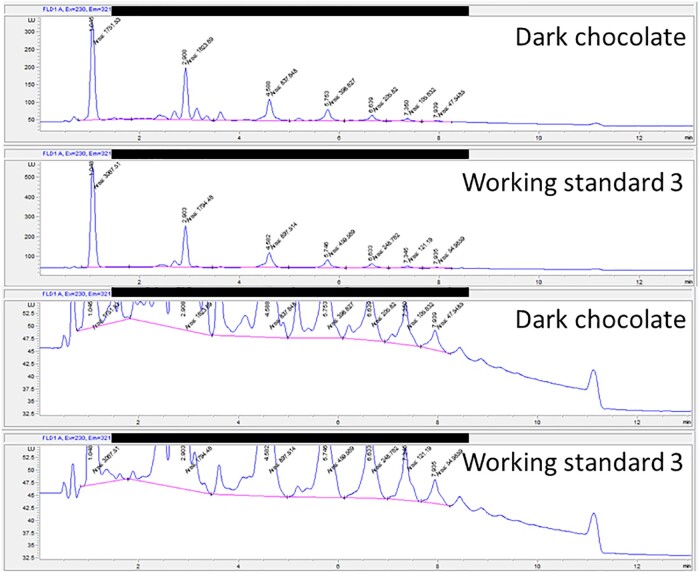
HPLC traces showing DP 1–7. Sample was run with an Agilent 1290 system at a PMT setting of 13. Valley-to-valley integration is shown. The main point of this figure is to highlight integration format in the working standard and a sample (here showing dark chocolate).

In the literature, two approaches for integration with HPLC methods have been reported, one integrating the complete baseline for the entire run, the other integrating the individual peaks valley-to-valley. R_s_ is clear for cocoa and chocolate samples, and a valley-to-valley integration approach was determined to be reproducible and robust in earlier method development steps. *See*Figure 2 for an example. Additionally, since there is more than one species under each DP peak, and there can be moderate resolution of isomers of the procyanidins in one DP peak, providing visual guidance in the figure ensures reproducibility.

Quantification and calculations:

Plot the concentration of each DPn (C_*DPn*_ from Equation 1) on the *x*-axis and the corresponding FLD peak area (FLD Area_DPn_) on the *y*-axis. This should result in seven separate standard curves (DP1–7). Use linear regression to obtain the slope (*m*_DPn_) and intercept *b*_DPn_ for each DPn standard curve. For each unknown sample, the mass fraction of cocoa flavanols in the sample (CF_DPn_; flavanols + procyanidins) is then calculated using Equation 2:

(2) $${\mathrm{CF}}_{\mathrm{DPn}}\;\left(\mathrm{mg}/g\;\mathrm{unk}\;\mathrm{sample}\right)=\frac{\left({\mathrm{FLD}\;\mathrm{Area}}_{\mathrm{DPn}}\;–\;b_{\mathrm{DPn}}\right)}{m_{\mathrm{DPn}}}\times \frac{{DV}_{\mathrm{Unk}}}{{\mathrm{Weight}}_{\mathrm{Unk}\;}(g)}\times \frac{100-\;\%\mathrm{fat}}{100}$$

where Weight_Unk_ and DV_Unk_ refer to the weight and the dilution volume used to prepare the unknown sample (*see*Table 3). A correction for the fat content (%) of the sample is included in the measurement.

The total cocoa flavanol in the defatted unknown sample is the sum of the individual CF_DPn_ values obtained from Equation 3:

(3) $$\mathrm{Total}\;\mathrm{CF}\;\left(\mathrm{mg}/g\right)=\sum_{\mathrm{DPn}=1}^{7}{\mathrm{CF}}_{\mathrm{DPn}}$$

## Results and Discussion

### Collaborative Study Results

Data were collected between February and June 2021. Each laboratory followed AOAC Method **2020.05** and determined a practice sample (NIST baking chocolate RM 8403). Eleven of the 12 participating laboratories were able to successfully determine fat content (51.7 ± 1.6%; mean ± standard deviation) and total CF (7.5 ± 0.4 mg/g; mean ± standard deviation). These 11 laboratories were instructed to move forward with the analysis of the remaining 14 samples. Of these remaining labs, only 10 successfully returned results as one laboratory faced technical challenges (not related to the method) and could not complete the study. Therefore, data from only 10 laboratories are presented as the final precision test results in this article.

Table 4 shows the total CF contents reported by 10 laboratories for 14 samples (seven matrixes in blind duplicates). The complete data set, including individual laboratory results for each degree of polymerization and system suitability data, is provided as Supplemental Information. Statistical analyses were performed according to AOAC guidelines for collaborative studies and multi-laboratory validations (19). Cochran, Grubbs, and double Grubbs outliers were identified and excluded from the estimation of repeatability and reproducibility. Materials were determined at concentrations ranging from 1 to 500 mg/g, representing milk chocolate and cocoa extract, respectively. These values were used to estimate repeatability and reproducibility standard deviations (S_r_ and S_R_, respectively) and relative standard deviations (RSD_r_ and RSD_R_, respectively). These values are shown in Table 5 alongside HorRat scores.

**Table 4.** Summary of total CF content determined by 10 laboratories for 14 samples analyzed in singlicates (seven matrixes in blind duplicates). Concentration is corrected to include fat contents

Material	Individual laboratory results for total cocoa flavanol (DP1–7) in mg/g (with fat correction)									
	1	2	3	4	5	6	7	8	9	10
Milk chocolate 1	1.0	1.1	0.6	1.1	0.8	0.9	0.9	1.1	1.4	1.0
Milk chocolate 2	1.0	1.1	0.6	1.2	0.8	0.9	1.0	1.2	1.4	0.9
Baking chocolate 1	7.1	7.3	7.3	7.9	7.2	7.5	7.2	8.3	7.8^a^	8.8
Baking chocolate 2	7.1	7.7	7.4	8.2	7.3	7.6	7.3	8.2	6.6^a^	8.8
Cocoa liquor 1	17.3	17.2	16.2	20.2	14.2	16.9	18.2	18.1	17.9	25.9^a^^,b^
Cocoa liquor 2	16.7	17.7	15.5	20.1	14.3	17.1	18.8	18	17.9	20.4^a^^,b^
Cocoa powder 1	12.9	13.7	13.2	14.9	12.7	12.4	13.6	13.8	14.4	14.4
Cocoa powder 2	13.1	14.6	12.2	14.6	13.3	12.0	14.0	13.8	14.1	14.6
Ready-to-mix supplement 1	80.3	71.7^c^	82.5	80.6	86.8	124.0^c^	87.4	72.2	87.4	88.1
Ready-to-mix supplement 2	80.8	73.1^c^	85.0	81.7	79.8	121.6^c^	81.4	79.1	87.5	91.4
Capsule supplement 1	480.9	424.0	539.2	533.1	497.1	487.4	543.0	453.2	519.7	492.1
Capsule supplement 2	465.6	397.8	523.9	527.2	522.4	462.3	528.4	460.9	445.9	524.2
Cocoa extract 1	512.0	445.0	550.8	545.9	524.2	491.6	575.6	514.6	489.6	530.6
Cocoa extract 2	515.7	457.6	552.9	582.4	541.4	492.0	555.0	514.2	511.7	546.3

a Cochran outlier.

b Grubbs outlier.

c Double Grubbs outlier.

Repeatability was estimated by RSD_r_ and ranged from 2 to 5% on a whole sample (fat-corrected, including the defatting step) for all product types. Thus, repeatability performances achieved the expectation set by AOAC SMPR 2012.001 (RSD_r_ ≤6% for 0.05–500 mg/g) (20). In addition, the repeatability performances represented a marked improvement from the previous AOAC CF methodology (AOAC *Official Method of Analysis* **2012.24**) (21), where RSD_r_ ranged from 4.2 to 9.6% for similar product types, all reported on a defatted material basis.

RSD_R_ was estimated between 6 and 10% for all but the milk chocolate sample. These reproducibility performances encompassed the entire methodology, including the material defatting step that can contribute to method variability. AOAC SMPR 2012.001 targeted RSD_R_ at ≤8%. This performance expectation was met for cocoa powder, cocoa extract, dietary supplement ready-to-mix powder, and baking chocolate. RSD_R_ slightly exceeded AOAC SMPR 2012.001 target performances for cocoa liquor and dietary supplement capsules (RSD_R_ ≤10% for both). RSD_R_ was determined at 22% for milk chocolate, while RSD_r_ and RSD_R_ on the defatted milk chocolate were 2.0 and 8.1%, respectively, highlighting both the reproducibility of the defatting step and the challenge to measure cocoa flavanol and procyanidins at very low concentrations. Similar to RSD_r_, RSD_R_ showed significant improvement compared to the previous AOAC Method **2012.24** methodology, which showed RSD_R_ ranging from 5 to 18% on defatted materials. It should be noted that the CF content determined in milk chocolate highlights the irrelevance of this matrix as a meaningful source of CF. However, the assessment of milk chocolate might still be relevant to specific application (e.g., document levels and variability in the marketplace; estimation of dietary intake from common food sources) and the understanding of method limitations at low concentrations (<5 mg/g), which may be important to know in certain settings.

RSD_r_ and RSD_R_ results demonstrated that adequate precision was achieved by AOAC Method **2020.05** for the determination of flavanols and procyanidins in cocoa-based products at the single and multi-laboratory scales. Comparison of precision achieved at the repeatability and reproducibility scales was demonstrated using HorRat and reported in Table 5. HorRat scores ranged from 1.8 to 4.0 and were comparable to other methodologies dedicated to analytes with diverse and unspecified molecular structures such as lycopene (22).

**Table 5.** Summary of results for repeatability (RSD_r_) and reproducibility (RSD_R_) across multiple laboratories (*N* shows the number of laboratories used for each matrix and *n* the total number of analysis for each sample)

	Average, mg/g	Sr	RSD_r_, %	SR	RSD_R_, %	HorRat	No. of laboratories used	No. of replicates used
Milk chocolate	1.0	0.05	4.8	0.2	22.4	4.0	10	20
Baking chocolate	7.7	0.1	1.6	0.6	7.4	1.8	9	18
Cocoa liquor	17.3	0.3	1.6	1.7	9.7	2.6	9	18
Cocoa powder	13.6	0.4	2.7	0.9	6.5	1.7	10	20
Ready-to-mix dietary supplement powder	83.3	3.1	3.7	4.8	5.8	2.0	8	16
Dietary supplement capsules	491.4	21.5	4.4	42.3	8.6	3.9	10	20
Cocoa extract	522.5	12.2	2.3	36.6	7.0	3.2	10	20

### Collaborator Comments

Technical challenges while implementing the method were also reported. One laboratory observed significant baseline drift within each run. This was resolved by using a higher grade of acetic acid for the mobile phase. One laboratory failed system suitability during its first run, and this was associated with an inadequate refill of mobile phase during the run. This was resolved after sharing technical resources on the importance of system equilibration.

## Conclusions

Twelve laboratories were invited and accepted to participate in this collaborative study. Of these 12 labs, 11 labs successfully implemented the method, 10 of which completed the study protocol. Repeatability and reproducibility were estimated for AOAC Method **2020.05** for a wide range of concentrations and matrixes. Repeatability performances all met performance expectations set by AOAC SMPR 2012.001 (≤6%). Reproducibility performance met expectations set by AOAC Method **2012.001** in four matrixes (RSD_R_ at ≤8%) and slightly exceeded in two matrixes (RSD_R_ at ≤10%), while failing to meet performance expectations for milk chocolate. The generally poorer performance of milk chocolate is likely attributable to the very low CF concentration (approximately 1 mg/g). As a result, it is recommended to limit this method to products with mass fraction above 5 mg/g, limiting the application of this method to very low concentrations like the one observed in milk chocolate. Industrial, academic, and governmental laboratories across multiple countries successfully implemented AOAC Method **2020.05**, demonstrating method accessibility to various scientific sectors and geographical regions while documenting performances that meet or closely meet AOAC standard method performance requirements (SMPR 2012.001) for a range of cocoa-based products expected to be the most common commercial sources of cocoa flavanols.

## Supplementary Material

qsac007_Supplementary_Data
