# Supplementary material for: Determination of Cocoa Flavanols and Procyanidins (by Degree of Polymerization DP1-7) in Cocoa-Based Products by Hydrophilic Interaction Chromatography Coupled With Fluorescence Detection: Collaborative Study
Source: J AOAC Int. 2022 Jan 26;105(4):1060–8. doi: 10.1093/jaoacint/qsac007 (PMC9247699; doi:10.1093/jaoacint/qsac007)
Supplement: qsac007_Supplementary_Data [file qsac007_supplementary_data.docx]

**Supplementary information:**

**A collaborative multi-laboratory study for AOAC2020.05: Determination of cocoa flavanols and procyanidins (by degree of polymerization DP1-7) in cocoa-based products by hydrophilic interaction chromatography coupled with fluorescence detection**

**Individual lab results**

| Lab1 (mg/g) | DP1 | DP2 | DP3 | DP4 | DP5 | DP6 | DP7 | Total | %Fat |
| --- | --- | --- | --- | --- | --- | --- | --- | --- | --- |
| Milk Chocolate 1 | 0.2 | 0.2 | 0.2 | 0.1 | 0.1 | 0.1 | 0.0 | 0.8 | 39.4 |
| Milk Chocolate 2 | 0.2 | 0.2 | 0.2 | 0.1 | 0.1 | 0.1 | 0.0 | 0.8 | 37.5 |
| Baking Chocolate 1 | 0.9 | 1.2 | 1.1 | 0.9 | 0.7 | 0.5 | 0.4 | 5.7 | 59.0 |
| Baking Chocolate 2 | 0.9 | 1.2 | 1.2 | 0.9 | 0.7 | 0.6 | 0.3 | 5.9 | 58.5 |
| Cocoa liquor 1 | 3.2 | 2.8 | 2.6 | 2.0 | 1.6 | 1.1 | 0.8 | 14.2 | 61.1 |
| Cocoa Liquor 2 | 3.3 | 2.9 | 2.6 | 2.0 | 1.5 | 1.1 | 0.9 | 14.3 | 60.9 |
| Cocoa Powder 1 | 3.0 | 2.8 | 2.3 | 1.7 | 1.3 | 0.9 | 0.6 | 12.7 | 17.7 |
| Cocoa Powder 2 | 3.1 | 3.0 | 2.6 | 1.8 | 1.3 | 1.0 | 0.6 | 13.3 | 17.1 |
| Ready-to-mix supplement 1 | 14.2 | 15.2 | 16.4 | 14.8 | 12.8 | 8.2 | 5.1 | 86.8 | N/A |
| Ready-to-mix supplement 2 | 13.3 | 13.6 | 14.3 | 12.2 | 10.1 | 10.2 | 6.2 | 79.8 | N/A |
| Capsule supplement 1 | 115.5 | 79.3 | 86.1 | 71.9 | 61.3 | 44.7 | 38.3 | 497.1 | N/A |
| Capsule supplement 2 | 118.3 | 81.9 | 91.5 | 76.6 | 61.3 | 50.8 | 42.1 | 522.4 | N/A |
| Cocoa Extract 1 | 113.5 | 82.9 | 90.1 | 81.1 | 69.0 | 54.4 | 33.2 | 524.2 | N/A |
| Cocoa Extract 2 | 117.8 | 86.3 | 91.9 | 80.0 | 70.2 | 50.4 | 44.7 | 541.4 | N/A |

| Lab 2 (mg/g) | DP1 | DP2 | DP3 | DP4 | DP5 | DP6 | DP7 | Total | %Fat |
| --- | --- | --- | --- | --- | --- | --- | --- | --- | --- |
| Milk Chocolate 1 | 0.2 | 0.2 | 0.2 | 0.1 | 0.1 | 0.1 | 0.1 | 1.0 | 31.2 |
| Milk Chocolate 2 | 0.2 | 0.2 | 0.2 | 0.1 | 0.1 | 0.1 | 0.1 | 1.0 | 31.3 |
| Baking Chocolate 1 | 1.2 | 1.3 | 1.4 | 1.1 | 0.9 | 0.7 | 0.5 | 7.1 | 51.8 |
| Baking Chocolate 2 | 1.2 | 1.3 | 1.4 | 1.1 | 0.9 | 0.7 | 0.5 | 7.1 | 51.6 |
| Cocoa liquor 1 | 3.8 | 3.2 | 3.2 | 2.5 | 2.0 | 1.5 | 1.1 | 17.3 | 55.1 |
| Cocoa Liquor 2 | 3.7 | 3.0 | 3.1 | 2.4 | 1.9 | 1.5 | 1.1 | 16.7 | 55.9 |
| Cocoa Powder 1 | 3.2 | 2.6 | 2.4 | 1.7 | 1.3 | 1.0 | 0.7 | 12.9 | 11.2 |
| Cocoa Powder 2 | 3.2 | 2.6 | 2.5 | 1.8 | 1.3 | 1.0 | 0.8 | 13.1 | 11.1 |
| Ready-to-mix supplement 1 | 14.2 | 13.1 | 15.4 | 12.5 | 10.4 | 8.5 | 6.2 | 80.3 | N/A |
| Ready-to-mix supplement 2 | 14.3 | 13.3 | 15.6 | 12.6 | 10.4 | 8.3 | 6.3 | 80.8 | N/A |
| Capsule supplement 1 | 109.2 | 75.1 | 80.1 | 70.3 | 61.4 | 48.5 | 36.3 | 480.9 | N/A |
| Capsule supplement 2 | 107.3 | 73.6 | 77.9 | 68.1 | 59.0 | 46.3 | 33.5 | 465.6 | N/A |
| Cocoa Extract 1 | 113.6 | 82.9 | 88.0 | 74.5 | 64.9 | 51.3 | 36.8 | 512.0 | N/A |
| Cocoa Extract 2 | 114.6 | 83.7 | 89.2 | 75.4 | 64.5 | 49.6 | 38.8 | 515.7 | N/A |

| Lab 3 (mg/g) | DP1 | DP2 | DP3 | DP4 | DP5 | DP6 | DP7 | Total | %Fat |
| --- | --- | --- | --- | --- | --- | --- | --- | --- | --- |
| Milk Chocolate 1 | 0.2 | 0.2 | 0.2 | 0.1 | 0.1 | 0.1 | 0.1 | 0.9 | 31.1 |
| Milk Chocolate 2 | 0.2 | 0.2 | 0.2 | 0.1 | 0.1 | 0.1 | 0.1 | 1.0 | 31.0 |
| Baking Chocolate 1 | 1.4 | 1.3 | 1.2 | 1.2 | 1.0 | 0.7 | 0.5 | 7.2 | 51.6 |
| Baking Chocolate 2 | 1.4 | 1.3 | 1.2 | 1.1 | 1.0 | 0.8 | 0.5 | 7.3 | 51.6 |
| Cocoa liquor 1 | 4.3 | 3.3 | 3.2 | 2.6 | 2.1 | 1.5 | 1.1 | 18.2 | 54.1 |
| Cocoa Liquor 2 | 4.2 | 3.2 | 3.2 | 2.8 | 2.4 | 1.6 | 1.3 | 18.8 | 54.2 |
| Cocoa Powder 1 | 3.6 | 2.5 | 2.1 | 1.8 | 1.5 | 1.1 | 0.9 | 13.6 | 10.9 |
| Cocoa Powder 2 | 3.6 | 2.7 | 2.2 | 1.9 | 1.6 | 1.2 | 0.9 | 14.0 | 10.8 |
| Ready-to-mix supplement 1 | 15.6 | 15.2 | 15.4 | 13.4 | 11.3 | 9.1 | 7.5 | 87.4 | N/A |
| Ready-to-mix supplement 2 | 15.4 | 14.0 | 14.3 | 12.8 | 10.7 | 8.5 | 5.8 | 81.4 | N/A |
| Capsule supplement 1 | 121.1 | 84.6 | 91.7 | 78.4 | 69.5 | 57.9 | 39.8 | 543.0 | N/A |
| Capsule supplement 2 | 117.8 | 80.4 | 87.4 | 76.0 | 69.3 | 56.2 | 41.4 | 528.4 | N/A |
| Cocoa Extract 1 | 125.7 | 91.9 | 96.9 | 83.7 | 71.8 | 59.0 | 46.6 | 575.6 | N/A |
| Cocoa Extract 2 | 123.1 | 90.2 | 95.3 | 78.8 | 69.4 | 55.7 | 42.6 | 555.0 | N/A |

| Lab 4 (mg/g) | DP1 | DP2 | DP3 | DP4 | DP5 | DP6 | DP7 | Total | %Fat |
| --- | --- | --- | --- | --- | --- | --- | --- | --- | --- |
| Milk Chocolate 1 | 0.2 | 0.2 | 0.2 | 0.2 | 0.1 | 0.1 | 0.1 | 1.1 | 30.6 |
| Milk Chocolate 2 | 0.2 | 0.2 | 0.2 | 0.2 | 0.1 | 0.1 | 0.1 | 1.1 | 30.8 |
| Baking Chocolate 1 | 1.2 | 1.4 | 1.5 | 1.1 | 0.9 | 0.7 | 0.5 | 7.3 | 51.5 |
| Baking Chocolate 2 | 1.2 | 1.5 | 1.6 | 1.2 | 1.0 | 0.7 | 0.6 | 7.7 | 51.5 |
| Cocoa liquor 1 | 4.0 | 3.3 | 3.2 | 2.5 | 1.9 | 1.4 | 1.0 | 17.2 | 54.9 |
| Cocoa Liquor 2 | 4.0 | 3.4 | 3.3 | 2.5 | 1.9 | 1.5 | 1.1 | 17.7 | 54.7 |
| Cocoa Powder 1 | 3.3 | 2.8 | 2.6 | 1.9 | 1.3 | 1.0 | 0.9 | 13.7 | 11.1 |
| Cocoa Powder 2 | 3.5 | 3.0 | 2.8 | 2.0 | 1.5 | 1.1 | 0.9 | 14.6 | 10.7 |
| Ready-to-mix supplement 1 | 12.9 | 12.0 | 13.2 | 11.4 | 9.5 | 7.2 | 5.5 | 71.7 | N/A |
| Ready-to-mix supplement 2 | 13.4 | 12.3 | 13.8 | 11.6 | 8.5 | 7.5 | 5.9 | 73.1 | N/A |
| Capsule supplement 1 | 115.0 | 73.4 | 74.1 | 57.8 | 46.4 | 33.6 | 23.9 | 424.0 | N/A |
| Capsule supplement 2 | 111.2 | 70.1 | 69.6 | 53.8 | 42.4 | 29.6 | 21.2 | 397.8 | N/A |
| Cocoa Extract 1 | 104.8 | 75.1 | 79.4 | 64.5 | 52.1 | 39.0 | 30.1 | 445.0 | N/A |
| Cocoa Extract 2 | 108.5 | 76.5 | 81.5 | 65.9 | 54.6 | 40.6 | 29.9 | 457.6 | N/A |

| Lab 5 (mg/g) | DP1 | DP2 | DP3 | DP4 | DP5 | DP6 | DP7 | Total | %Fat |
| --- | --- | --- | --- | --- | --- | --- | --- | --- | --- |
| Milk Chocolate 1 | 0.2 | 0.2 | 0.2 | 0.1 | 0.1 | 0.1 | 0.1 | 0.9 | 31.1 |
| Milk Chocolate 2 | 0.2 | 0.2 | 0.2 | 0.1 | 0.1 | 0.1 | 0.0 | 0.9 | 31.3 |
| Baking Chocolate 1 | 1.4 | 1.3 | 1.5 | 1.1 | 0.9 | 0.7 | 0.5 | 7.5 | 52.1 |
| Baking Chocolate 2 | 1.4 | 1.3 | 1.5 | 1.1 | 0.9 | 0.7 | 0.5 | 7.6 | 51.8 |
| Cocoa liquor 1 | 4.0 | 3.3 | 3.2 | 2.3 | 1.8 | 1.4 | 1.0 | 16.9 | 55.3 |
| Cocoa Liquor 2 | 4.1 | 3.3 | 3.2 | 2.4 | 1.8 | 1.3 | 1.0 | 17.1 | 55.0 |
| Cocoa Powder 1 | 3.4 | 2.5 | 2.4 | 1.5 | 1.1 | 0.8 | 0.6 | 12.4 | 11.1 |
| Cocoa Powder 2 | 3.4 | 2.5 | 2.3 | 1.4 | 1.1 | 0.8 | 0.5 | 12.0 | 11.3 |
| Ready-to-mix supplement 1 | 21.3 | 19.4 | 22.8 | 19.9 | 16.9 | 13.3 | 10.4 | 124.0 | N/A |
| Ready-to-mix supplement 2 | 21.2 | 19.1 | 22.4 | 19.5 | 16.6 | 13.0 | 9.9 | 121.6 | N/A |
| Capsule supplement 1 | 111.4 | 77.8 | 83.3 | 71.6 | 61.9 | 46.8 | 34.5 | 487.4 | N/A |
| Capsule supplement 2 | 107.2 | 73.7 | 78.6 | 66.8 | 58.0 | 44.9 | 33.1 | 462.3 | N/A |
| Cocoa Extract 1 | 107.9 | 76.5 | 83.0 | 71.9 | 63.8 | 50.6 | 38.0 | 491.6 | N/A |
| Cocoa Extract 2 | 108.1 | 77.7 | 83.2 | 72.9 | 63.0 | 49.2 | 37.8 | 492.0 | N/A |

| Lab 6 (mg/g) | DP1 | DP2 | DP3 | DP4 | DP5 | DP6 | DP7 | Total | %Fat |
| --- | --- | --- | --- | --- | --- | --- | --- | --- | --- |
| Milk Chocolate 1 | 0.2 | 0.1 | 0.1 | 0.1 | 0.1 | 0.0 | 0.0 | 0.6 | 31.8 |
| Milk Chocolate 2 | 0.1 | 0.1 | 0.1 | 0.1 | 0.1 | 0.0 | 0.0 | 0.6 | 31.0 |
| Baking Chocolate 1 | 1.2 | 1.3 | 1.4 | 1.1 | 0.9 | 0.7 | 0.5 | 7.3 | 51.9 |
| Baking Chocolate 2 | 1.2 | 1.3 | 1.4 | 1.1 | 1.0 | 0.7 | 0.5 | 7.4 | 49.5 |
| Cocoa liquor 1 | 3.8 | 3.1 | 3.0 | 2.3 | 1.8 | 1.3 | 0.9 | 16.2 | 54.7 |
| Cocoa Liquor 2 | 3.7 | 3.0 | 2.9 | 2.2 | 1.7 | 1.2 | 0.9 | 15.5 | 55.1 |
| Cocoa Powder 1 | 3.4 | 2.8 | 2.4 | 1.7 | 1.3 | 1.0 | 0.7 | 13.2 | 10.0 |
| Cocoa Powder 2 | 3.1 | 2.6 | 2.2 | 1.6 | 1.2 | 0.9 | 0.6 | 12.2 | 10.9 |
| Ready-to-mix supplement 1 | 13.2 | 13.0 | 14.9 | 13.2 | 11.7 | 9.3 | 7.2 | 82.5 | N/A |
| Ready-to-mix supplement 2 | 13.5 | 13.2 | 15.4 | 13.7 | 12.1 | 9.7 | 7.4 | 85.0 | N/A |
| Capsule supplement 1 | 118.3 | 81.9 | 90.3 | 79.7 | 70.7 | 56.7 | 41.6 | 539.2 | N/A |
| Capsule supplement 2 | 113.6 | 79.3 | 87.6 | 78.1 | 69.4 | 54.8 | 41.1 | 523.9 | N/A |
| Cocoa Extract 1 | 115.8 | 86.4 | 93.3 | 82.5 | 72.1 | 56.7 | 44.0 | 550.8 | N/A |
| Cocoa Extract 2 | 116.7 | 86.8 | 93.0 | 81.9 | 72.1 | 57.7 | 44.7 | 552.9 | N/A |

| Lab 7 (mg/g) | DP1 | DP2 | DP3 | DP4 | DP5 | DP6 | DP7 | Total | %Fat |
| --- | --- | --- | --- | --- | --- | --- | --- | --- | --- |
| Milk Chocolate 1 | 0.2 | 0.2 | 0.2 | 0.2 | 0.1 | 0.1 | 0.1 | 1.1 | 28.8 |
| Milk Chocolate 2 | 0.2 | 0.2 | 0.2 | 0.2 | 0.2 | 0.1 | 0.1 | 1.2 | 28.8 |
| Baking Chocolate 1 | 1.2 | 1.3 | 1.5 | 1.3 | 1.1 | 0.8 | 0.6 | 7.9 | 50.0 |
| Baking Chocolate 2 | 1.3 | 1.4 | 1.6 | 1.4 | 1.2 | 0.8 | 0.6 | 8.2 | 49.3 |
| Cocoa liquor 1 | 4.4 | 3.6 | 3.6 | 3.1 | 2.5 | 1.8 | 1.2 | 20.2 | 52.4 |
| Cocoa Liquor 2 | 4.3 | 3.5 | 3.6 | 3.1 | 2.6 | 1.8 | 1.2 | 20.1 | 52.5 |
| Cocoa Powder 1 | 3.7 | 2.9 | 2.7 | 2.1 | 1.7 | 1.1 | 0.8 | 14.9 | 7.2 |
| Cocoa Powder 2 | 3.6 | 2.8 | 2.7 | 2.1 | 1.6 | 1.1 | 0.8 | 14.6 | 8.0 |
| Ready-to-mix supplement 1 | 13.5 | 13.4 | 14.7 | 12.9 | 11.0 | 8.7 | 6.4 | 80.6 | N/A |
| Ready-to-mix supplement 2 | 13.6 | 13.2 | 14.7 | 13.0 | 11.2 | 9.0 | 6.9 | 81.7 | N/A |
| Capsule supplement 1 | 120.8 | 82.8 | 88.5 | 79.3 | 70.1 | 52.7 | 39.0 | 533.1 | N/A |
| Capsule supplement 2 | 119.8 | 82.3 | 86.7 | 77.6 | 69.3 | 52.7 | 38.8 | 527.2 | N/A |
| Cocoa Extract 1 | 121.0 | 89.6 | 90.5 | 80.6 | 70.6 | 54.4 | 39.2 | 545.9 | N/A |
| Cocoa Extract 2 | 127.9 | 94.6 | 95.7 | 86.9 | 74.8 | 59.1 | 43.5 | 582.4 | N/A |

| Lab 8 (mg/g) | DP1 | DP2 | DP3 | DP4 | DP5 | DP6 | DP7 | Total | %Fat |
| --- | --- | --- | --- | --- | --- | --- | --- | --- | --- |
| Milk Chocolate 1 | 0.3 | 0.3 | 0.3 | 0.2 | 0.2 | 0.1 | 0.1 | 1.4 | 31.3 |
| Milk Chocolate 2 | 0.3 | 0.3 | 0.3 | 0.2 | 0.2 | 0.1 | 0.1 | 1.4 | 31.4 |
| Baking Chocolate 1 | 1.4 | 1.4 | 1.6 | 1.2 | 1.0 | 0.8 | 0.6 | 7.8 | 53.2 |
| Baking Chocolate 2 | 1.2 | 1.2 | 1.3 | 1.0 | 0.8 | 0.6 | 0.5 | 6.6 | 53.1 |
| Cocoa liquor 1 | 4.1 | 3.2 | 3.3 | 2.6 | 2.0 | 1.5 | 1.1 | 17.9 | 55.0 |
| Cocoa Liquor 2 | 4.1 | 3.1 | 3.3 | 2.6 | 2.1 | 1.5 | 1.1 | 17.9 | 55.5 |
| Cocoa Powder 1 | 3.9 | 2.8 | 2.7 | 1.9 | 1.4 | 1.0 | 0.8 | 14.4 | 11.0 |
| Cocoa Powder 2 | 3.8 | 2.7 | 2.7 | 1.8 | 1.4 | 1.0 | 0.8 | 14.1 | 11.3 |
| Ready-to-mix supplement 1 | 16.4 | 14.1 | 16.1 | 13.4 | 11.3 | 8.8 | 7.3 | 87.4 | N/A |
| Ready-to-mix supplement 2 | 15.3 | 14.0 | 16.5 | 14.0 | 12.0 | 9.2 | 6.6 | 87.5 | N/A |
| Capsule supplement 1 | 117.6 | 79.3 | 88.3 | 75.2 | 65.9 | 53.9 | 39.5 | 519.7 | N/A |
| Capsule supplement 2 | 100.9 | 68.2 | 75.7 | 63.0 | 56.3 | 46.1 | 35.7 | 445.9 | N/A |
| Cocoa Extract 1 | 110.5 | 77.6 | 85.4 | 72.3 | 62.3 | 46.3 | 35.1 | 489.6 | N/A |
| Cocoa Extract 2 | 115.4 | 79.8 | 89.3 | 72.8 | 63.8 | 49.8 | 40.9 | 511.7 | N/A |

| Lab 9 (mg/g) | DP1 | DP2 | DP3 | DP4 | DP5 | DP6 | DP7 | Total | %Fat |
| --- | --- | --- | --- | --- | --- | --- | --- | --- | --- |
| Milk Chocolate 1 | 0.2 | 0.2 | 0.2 | 0.2 | 0.1 | 0.1 | 0.1 | 1.1 | 30.8 |
| Milk Chocolate 2 | 0.2 | 0.2 | 0.2 | 0.2 | 0.1 | 0.1 | 0.1 | 1.2 | 31.3 |
| Baking Chocolate 1 | 1.3 | 1.5 | 1.6 | 1.3 | 1.1 | 0.9 | 0.7 | 8.3 | 51.6 |
| Baking Chocolate 2 | 1.2 | 1.6 | 1.6 | 1.2 | 1.0 | 0.9 | 0.6 | 8.2 | 51.8 |
| Cocoa liquor 1 | 4.1 | 3.5 | 3.4 | 2.7 | 2.1 | 1.4 | 1.1 | 18.1 | 54.6 |
| Cocoa Liquor 2 | 4.0 | 3.5 | 3.3 | 2.6 | 2.0 | 1.5 | 1.1 | 18.0 | 54.8 |
| Cocoa Powder 1 | 3.4 | 3.0 | 2.6 | 1.8 | 1.3 | 0.9 | 0.7 | 13.8 | 11.0 |
| Cocoa Powder 2 | 3.4 | 3.0 | 2.6 | 1.8 | 1.3 | 1.0 | 0.7 | 13.8 | 11.0 |
| Ready-to-mix supplement 1 | 13.4 | 12.0 | 13.1 | 11.2 | 9.7 | 7.3 | 5.4 | 72.2 | N/A |
| Ready-to-mix supplement 2 | 14.1 | 12.7 | 14.3 | 12.5 | 10.5 | 8.6 | 6.3 | 79.1 | N/A |
| Capsule supplement 1 | 103.4 | 70.4 | 79.0 | 67.5 | 56.6 | 43.6 | 32.7 | 453.2 | N/A |
| Capsule supplement 2 | 108.6 | 72.7 | 82.9 | 69.9 | 55.1 | 41.4 | 30.3 | 460.9 | N/A |
| Cocoa Extract 1 | 111.6 | 82.6 | 90.8 | 76.5 | 62.7 | 52.0 | 38.4 | 514.6 | N/A |
| Cocoa Extract 2 | 110.9 | 83.8 | 89.9 | 77.7 | 64.0 | 48.0 | 39.8 | 514.2 | N/A |

| Lab 10 (mg/g) | DP1 | DP2 | DP3 | DP4 | DP5 | DP6 | DP7 | Total | %Fat |
| --- | --- | --- | --- | --- | --- | --- | --- | --- | --- |
| Milk Chocolate 1 | 0.2 | 0.2 | 0.2 | 0.2 | 0.1 | 0.1 | 0.1 | 1.0 | 31.1 |
| Milk Chocolate 2 | 0.2 | 0.2 | 0.2 | 0.1 | 0.1 | 0.1 | 0.1 | 0.9 | 31.1 |
| Baking Chocolate 1 | 1.3 | 1.6 | 1.7 | 1.4 | 1.2 | 0.9 | 0.8 | 8.8 | 51.4 |
| Baking Chocolate 2 | 1.3 | 1.6 | 1.7 | 1.3 | 1.2 | 0.9 | 0.8 | 8.8 | 51.6 |
| Cocoa liquor 1 | 4.2 | 3.8 | 3.7 | 8.3 | 2.5 | 1.9 | 1.4 | 25.9 | 54.0 |
| Cocoa Liquor 2 | 4.3 | 3.7 | 3.7 | 3.0 | 2.4 | 1.9 | 1.4 | 20.4 | 53.8 |
| Cocoa Powder 1 | 3.3 | 2.8 | 2.6 | 1.9 | 1.6 | 1.2 | 1.0 | 14.4 | 10.7 |
| Cocoa Powder 2 | 3.4 | 2.9 | 2.6 | 1.9 | 1.6 | 1.2 | 0.9 | 14.6 | 10.9 |
| Ready-to-mix supplement 1 | 13.7 | 14.7 | 16.1 | 14.4 | 12.3 | 9.8 | 7.1 | 88.1 | N/A |
| Ready-to-mix supplement 2 | 14.1 | 15.2 | 16.8 | 14.8 | 12.6 | 10.0 | 8.1 | 91.4 | N/A |
| Capsule supplement 1 | 112.2 | 82.1 | 84.1 | 70.9 | 60.2 | 46.9 | 35.7 | 492.1 | N/A |
| Capsule supplement 2 | 117.2 | 85.3 | 87.0 | 75.2 | 64.9 | 51.6 | 43.1 | 524.2 | N/A |
| Cocoa Extract 1 | 111.5 | 87.9 | 90.3 | 77.4 | 66.9 | 53.5 | 43.1 | 530.6 | N/A |
| Cocoa Extract 2 | 113.4 | 90.0 | 92.0 | 78.6 | 70.2 | 56.7 | 45.4 | 546.3 | N/A |

**Individual lab system suitability**

| Lab 1 - sequence #1 | DP1 | DP2 | DP3 | DP4 | DP5 | DP6 | DP7 | DP1-7 |
| --- | --- | --- | --- | --- | --- | --- | --- | --- |
| R^2^ | 1.00 | 1.00 | 0.99 | 0.99 | 0.99 | 0.99 | 0.99 | n/a |
| System suit. sample area (%RSD) | 1.83 | 1.62 | 1.46 | 3.34 | 1.34 | 1.81 | 6.26 | n/a |
| System suit. sample ret. time (%RSD) | 0.84 | 0.74 | 0.52 | 0.44 | 0.42 | 0.43 | 0.39 | n/a |
| Recovery (%) | n/a | n/a | n/a | n/a | n/a | n/a | n/a | 107.3 |
| Bracketing std. #1 area. rel. dev. (%) | 0.83 | 1.73 | 2.87 | 4.14 | 9.48 | 2.58 | 10.42 | n/a |
| Bracketing std. #1 ret. time rec. (%) | 104.96 | 104.22 | 102.11 | 101.50 | 101.20 | 101.04 | 100.89 | n/a |
| Bracketing std. #2 rel. dev. (%) | 1.26 | 4.36 | 3.25 | 4.70 | 9.43 | 13.47 | 13.34 | n/a |
| Bracketing std. #2 ret. time rec. (%) | 107.11 | 104.92 | 102.42 | 101.79 | 101.44 | 101.21 | 101.05 | n/a |

| Lab 1 - sequence #2 | DP1 | DP2 | DP3 | DP4 | DP5 | DP6 | DP7 | DP1-7 |
| --- | --- | --- | --- | --- | --- | --- | --- | --- |
| R^2^ | 1.00 | 1.00 | 1.00 | 1.00 | 1.00 | 0.99 | 0.99 | n/a |
| System suit. sample area (%RSD) | 1.70 | 1.64 | 1.32 | 1.89 | 3.14 | 4.93 | 6.18 | n/a |
| System suit. sample ret. time (%RSD) | 0.48 | 0.36 | 0.18 | 0.15 | 0.15 | 0.14 | 0.14 | n/a |
| Recovery (%) | n/a | n/a | n/a | n/a | n/a | n/a | n/a | 90.7 |
| Bracketing std. #1 area. rel. dev. (%) | 1.35 | 0.53 | 2.25 | 1.39 | 1.28 | 0.44 | 3.29 | n/a |
| Bracketing std. #1 ret. time rec. (%) | 102.63 | 101.72 | 101.24 | 101.13 | 101.00 | 100.92 | 100.90 | n/a |
| Bracketing std. #2 rel. dev. (%) | 4.57 | 4.24 | 1.53 | 1.47 | 8.12 | 2.11 | 2.75 | n/a |
| Bracketing std. #2 ret. time rec. (%) | 107.20 | 104.97 | 103.52 | 102.93 | 102.48 | 102.15 | 101.92 | n/a |

| Lab 1 - sequence #3 | DP1 | DP2 | DP3 | DP4 | DP5 | DP6 | DP7 | DP1-7 |
| --- | --- | --- | --- | --- | --- | --- | --- | --- |
| R^2^ | 1.00 | 1.00 | 1.00 | 1.00 | 0.99 | 1.00 | 1.00 | n/a |
| System suit. sample area (%RSD) | 0.67 | 1.86 | 2.93 | 1.41 | 5.56 | 1.33 | 5.76 | n/a |
| System suit. sample ret. time (%RSD) | 0.80 | 0.57 | 0.42 | 0.42 | 0.40 | 0.38 | 0.34 | n/a |
| Recovery (%) | n/a | n/a | n/a | n/a | n/a | n/a | n/a | 95.7 |
| Bracketing std. #1 area. rel. dev. (%) | 0.76 | 2.04 | 4.51 | 2.40 | 5.81 | 1.97 | 0.56 | n/a |
| Bracketing std. #1 ret. time rec. (%) | 101.94 | 101.18 | 100.83 | 100.67 | 100.55 | 100.51 | 100.45 | n/a |
| Bracketing std. #2 rel. dev. (%) | 3.43 | 3.82 | 4.03 | 2.74 | 4.77 | 4.99 | 2.42 | n/a |
| Bracketing std. #2 ret. time rec. (%) | 95.43 | 95.41 | 97.30 | 97.85 | 98.18 | 98.40 | 98.56 | n/a |

| Lab 2 | DP1 | DP2 | DP3 | DP4 | DP5 | DP6 | DP7 | DP1-7 |
| --- | --- | --- | --- | --- | --- | --- | --- | --- |
| R^2^ | 1.00 | 1.00 | 1.00 | 1.00 | 1.00 | 1.00 | 0.99 | n/a |
| System suit. sample area (%RSD) | 0.19 | 0.43 | 0.24 | 0.46 | 1.15 | 1.62 | 2.09 | n/a |
| System suit. sample ret. time (%RSD) | 0.00 | 0.15 | 0.12 | 0.00 | 0.00 | 0.00 | 0.00 | n/a |
| Recovery (%) | n/a | n/a | n/a | n/a | n/a | n/a | n/a | 96.8 |
| Bracketing std. #1 area. rel. dev. (%) | 0.76 | 0.08 | 1.03 | 2.26 | 7.20 | 8.73 | 9.48 | n/a |
| Bracketing std. #1 ret. time rec. (%) | 100.00 | 99.93 | 99.87 | 100.00 | 99.85 | 100.00 | 99.87 | n/a |
| Bracketing std. #2 rel. dev. (%) | 0.34 | 0.64 | 0.01 | 0.71 | 1.13 | 0.78 | 3.38 | n/a |
| Bracketing std. #2 ret. time rec. (%) | 97.81 | 101.59 | 101.24 | 101.34 | 101.36 | 101.44 | 101.59 | n/a |

| Lab 3 - sequence #1 | DP1 | DP2 | DP3 | DP4 | DP5 | DP6 | DP7 | DP1-7 |
| --- | --- | --- | --- | --- | --- | --- | --- | --- |
| R^2^ | 1.00 | 1.00 | 1.00 | 1.00 | 1.00 | 1.00 | 0.99 | n/a |
| System suit. sample area (%RSD) | 0.82 | 1.90 | 2.08 | 3.42 | 2.03 | 1.71 | 1.27 | n/a |
| System suit. sample ret. time (%RSD) | 0.31 | 0.08 | 0.07 | 0.07 | 0.07 | 0.06 | 0.07 | n/a |
| Recovery (%) | n/a | n/a | n/a | n/a | n/a | n/a | n/a | 101.3 |
| Bracketing std. #1 area. rel. dev. (%) | 3.96 | 3.69 | 2.88 | 1.95 | 2.25 | 1.04 | 0.95 | n/a |
| Bracketing std. #1 ret. time rec. (%) | 99.94 | 100.07 | 100.04 | 99.99 | 99.95 | 99.94 | 99.94 | n/a |
| Bracketing std. #2 rel. dev. (%) | 2.63 | 1.32 | 0.24 | 0.90 | 3.40 | 7.32 | 3.33 | n/a |
| Bracketing std. #2 ret. time rec. (%) | 99.94 | 100.04 | 99.99 | 99.94 | 99.92 | 99.95 | 99.94 | n/a |

| Lab 3 - sequence #2 | DP1 | DP2 | DP3 | DP4 | DP5 | DP6 | DP7 | DP1-7 |
| --- | --- | --- | --- | --- | --- | --- | --- | --- |
| R^2^ | 1.00 | 1.00 | 1.00 | 1.00 | 1.00 | 1.00 | 1.00 | n/a |
| System suit. sample area (%RSD) | 1.10 | 0.73 | 0.53 | 1.72 | 1.99 | 1.90 | 4.37 | n/a |
| System suit. sample ret. time (%RSD) | 0.26 | 0.14 | 0.03 | 0.03 | 0.04 | 0.04 | 0.04 | n/a |
| Recovery (%) | n/a | n/a | n/a | n/a | n/a | n/a | n/a | 102.2 |
| Bracketing std. #1 area. rel. dev. (%) | 2.82 | 3.49 | 1.08 | 0.05 | 0.09 | 2.30 | 6.58 | n/a |
| Bracketing std. #1 ret. time rec. (%) | 99.90 | 100.00 | 99.98 | 99.94 | 99.95 | 99.94 | 99.97 | n/a |

| Lab 4 | DP1 | DP2 | DP3 | DP4 | DP5 | DP6 | DP7 | DP1-7 |
| --- | --- | --- | --- | --- | --- | --- | --- | --- |
| R^2^ | 1.00 | 1.00 | 1.00 | 1.00 | 1.00 | 1.00 | 1.00 | n/a |
| System suit. sample area (%RSD) | 0.59 | 0.64 | 1.19 | 0.79 | 1.75 | 1.69 | 0.94 | n/a |
| System suit. sample ret. time (%RSD) | 0.13 | 0.15 | 0.08 | 0.04 | 0.09 | 0.02 | 0.03 | n/a |
| Recovery (%) | n/a | n/a | n/a | n/a | n/a | n/a | n/a | 101.1 |
| Bracketing std. #1 area. rel. dev. (%) | 0.86 | 0.28 | 0.60 | 2.52 | 0.27 | 1.12 | 2.24 | n/a |
| Bracketing std. #1 ret. time rec. (%) | 100.21 | 100.20 | 99.80 | 100.05 | 100.02 | 100.07 | 100.06 | n/a |
| Bracketing std. #2 rel. dev. (%) | 1.99 | 2.62 | 4.89 | 4.82 | 7.26 | 8.96 | 11.12 | n/a |
| Bracketing std. #2 ret. time rec. (%) | 100.40 | 100.16 | 99.59 | 99.95 | 100.11 | 100.12 | 100.05 | n/a |

| Lab 5 - sequence #1 | DP1 | DP2 | DP3 | DP4 | DP5 | DP6 | DP7 | DP1-7 |
| --- | --- | --- | --- | --- | --- | --- | --- | --- |
| R^2^ | 1.00 | 1.00 | 1.00 | 1.00 | 1.00 | 1.00 | 0.99 | n/a |
| System suit. sample area (%RSD) | 0.56 | 1.17 | 1.18 | 1.49 | 1.79 | 1.83 | 2.61 | n/a |
| System suit. sample ret. time (%RSD) | 0.30 | 0.07 | 0.09 | 0.03 | 0.06 | 0.06 | 0.03 | n/a |
| Recovery (%) | n/a | n/a | n/a | n/a | n/a | n/a | n/a | 94.2 |
| Bracketing std. #1 area. rel. dev. (%) | 3.10 | 3.30 | 4.40 | 3.70 | 0.50 | 0.10 | 0.10 | n/a |
| Bracketing std. #1 ret. time rec. (%) | 100.40 | 99.30 | 100.20 | 99.90 | 100.00 | 100.00 | 100.10 | n/a |
| Bracketing std. #2 rel. dev. (%) | 3.60 | 3.80 | 4.40 | 4.00 | 0.80 | 0.60 | 1.20 | n/a |
| Bracketing std. #2 ret. time rec. (%) | 100.40 | 100.10 | 100.20 | 100.10 | 100.20 | 100.10 | 100.10 | n/a |

| Lab 5 - sequence #2 | DP1 | DP2 | DP3 | DP4 | DP5 | DP6 | DP7 | DP1-7 |
| --- | --- | --- | --- | --- | --- | --- | --- | --- |
| R^2^ | 1.00 | 1.00 | 1.00 | 1.00 | 1.00 | 1.00 | 0.99 | n/a |
| System suit. sample area (%RSD) | 0.94 | 0.33 | 0.98 | 1.28 | 0.98 | 1.75 | 1.73 | n/a |
| System suit. sample ret. time (%RSD) | 1.00 | 1.00 | 1.00 | 1.00 | 1.00 | 1.00 | 0.99 | n/a |
| Recovery (%) | n/a | n/a | n/a | n/a | n/a | n/a | n/a | 101.2 |
| Bracketing std. #1 area. rel. dev. (%) | 4.10 | 3.30 | 4.40 | 4.90 | 3.80 | 1.80 | 1.40 | n/a |
| Bracketing std. #1 ret. time rec. (%) | 98.10 | 99.60 | 99.90 | 100.00 | 100.10 | 100.10 | 100.10 | n/a |

| Lab 6 - sequence #1 | DP1 | DP2 | DP3 | DP4 | DP5 | DP6 | DP7 | DP1-7 |
| --- | --- | --- | --- | --- | --- | --- | --- | --- |
| R^2^ | 1.00 | 1.00 | 1.00 | 1.00 | 1.00 | 1.00 | 1.00 | n/a |
| System suit. sample area (%RSD) | 0.32 | 0.21 | 0.37 | 0.48 | 1.14 | 0.82 | 1.40 | n/a |
| System suit. sample ret. time (%RSD) | 0.05 | 0.05 | 0.03 | 0.02 | 0.03 | 0.02 | 0.01 | n/a |
| Recovery (%) | n/a | n/a | n/a | n/a | n/a | n/a | n/a | 97.8 |
| Bracketing std. #1 area. rel. dev. (%) | 1.85 | 1.54 | 1.65 | 3.43 | 4.48 | 6.47 | 7.89 | n/a |
| Bracketing std. #1 ret. time rec. (%) | 100.08 | 100.23 | 100.05 | 100.05 | 100.01 | 100.01 | 99.98 | n/a |
| Bracketing std. #2 rel. dev. (%) | 3.10 | 0.75 | 0.09 | 2.08 | 3.40 | 4.42 | 5.85 | n/a |
| Bracketing std. #2 ret. time rec. (%) | 100.19 | 99.97 | 99.92 | 99.95 | 99.97 | 99.96 | 99.93 | n/a |

| Lab 6 - sequence #2 | DP1 | DP2 | DP3 | DP4 | DP5 | DP6 | DP7 | DP1-7 |
| --- | --- | --- | --- | --- | --- | --- | --- | --- |
| R^2^ | 1.00 | 1.00 | 1.00 | 1.00 | 1.00 | 1.00 | 1.00 | n/a |
| System suit. sample area (%RSD) | 0.19 | 0.28 | 0.61 | 0.38 | 1.08 | 1.85 | 2.31 | n/a |
| System suit. sample ret. time (%RSD) | 0.06 | 0.05 | 0.01 | 0.01 | 0.02 | 0.01 | 0.01 | n/a |
| Recovery (%) | n/a | n/a | n/a | n/a | n/a | n/a | n/a | 102.3 |
| Bracketing std. #1 area. rel. dev. (%) | 2.05 | 2.32 | 2.43 | 3.60 | 6.83 | 7.73 | 8.72 | n/a |
| Bracketing std. #1 ret. time rec. (%) | 99.61 | 99.66 | 99.79 | 99.91 | 99.92 | 99.91 | 99.91 | n/a |
| Bracketing std. #2 rel. dev. (%) | 1.99 | 3.20 | 3.95 | 4.55 | 5.79 | 7.15 | 8.60 | n/a |
| Bracketing std. #2 ret. time rec. (%) | 99.40 | 99.28 | 99.56 | 99.80 | 99.80 | 99.81 | 99.78 | n/a |

| Lab 7 | DP1 | DP2 | DP3 | DP4 | DP5 | DP6 | DP7 | DP1-7 |
| --- | --- | --- | --- | --- | --- | --- | --- | --- |
| R^2^ | 1.00 | 1.00 | 1.00 | 1.00 | 1.00 | 1.00 | 1.00 | n/a |
| System suit. sample area (%RSD) | 0.60 | 0.40 | 0.85 | 2.79 | 2.22 | 2.56 | 3.34 | n/a |
| System suit. sample ret. time (%RSD) | 0.56 | 0.21 | 0.13 | 0.11 | 0.10 | 0.09 | 0.07 | n/a |
| Recovery (%) | n/a | n/a | n/a | n/a | n/a | n/a | n/a | 100.3 |
| Bracketing std. #1 area. rel. dev. (%) | 1.39 | 0.18 | 0.56 | 12.50 | 11.28 | 3.93 | 2.92 | n/a |
| Bracketing std. #1 ret. time rec. (%) | 100.25 | 100.23 | 100.14 | 100.12 | 100.07 | 100.10 | 99.88 | n/a |
| Bracketing std. #2 rel. dev. (%) | 4.57 | 3.26 | 1.07 | 11.83 | 17.82 | 9.73 | 3.21 | n/a |
| Bracketing std. #2 ret. time rec. (%) | 100.25 | 99.84 | 99.90 | 99.92 | 99.89 | 99.77 | 99.73 | n/a |

| Lab 8 | DP1 | DP2 | DP3 | DP4 | DP5 | DP6 | DP7 | DP1-7 |
| --- | --- | --- | --- | --- | --- | --- | --- | --- |
| R^2^ | 0.99 | 0.99 | 0.99 | 0.99 | 0.99 | 0.99 | 0.99 | n/a |
| System suit. sample area (%RSD) | 0.88 | 0.27 | 1.19 | 0.63 | 1.82 | 0.95 | 1.68 | n/a |
| System suit. sample ret. time (%RSD) | 0.00 | 0.00 | 0.08 | 0.00 | 0.06 | 0.00 | 0.00 | n/a |
| Recovery (%) | n/a | n/a | n/a | n/a | n/a | n/a | n/a | 98.3 |
| Bracketing std. #1 area. rel. dev. (%) | 3.53 | 0.73 | 3.40 | 0.41 | 2.59 | 3.12 | 6.18 | n/a |
| Bracketing std. #1 ret. time rec. (%) | 100.00 | 100.00 | 100.04 | 100.00 | 99.97 | 100.00 | 100.00 | n/a |
| Bracketing std. #2 rel. dev. (%) | 1.86 | 4.95 | 1.20 | 3.74 | 1.10 | 1.91 | 3.49 | n/a |
| Bracketing std. #2 ret. time rec. (%) | 100.00 | 100.00 | 100.21 | 100.00 | 100.10 | 100.12 | 100.00 | n/a |

| Lab 9 - sequence #1 | DP1 | DP2 | DP3 | DP4 | DP5 | DP6 | DP7 | DP1-7 |
| --- | --- | --- | --- | --- | --- | --- | --- | --- |
| R^2^ | 1.00 | 1.00 | 1.00 | 1.00 | 1.00 | 1.00 | 1.00 | n/a |
| System suit. sample area (%RSD) | 1.11 | 0.88 | 1.40 | 2.25 | 1.97 | 1.40 | 6.12 | n/a |
| System suit. sample ret. time (%RSD) | 0.21 | 0.22 | 0.08 | 0.04 | 0.04 | 0.02 | 0.04 | n/a |
| Recovery (%) | n/a | n/a | n/a | n/a | n/a | n/a | n/a | 101.2 |
| Bracketing std. #1 area. rel. dev. (%) | 2.09 | 1.26 | 3.20 | 4.37 | 0.83 | 3.99 | 6.64 | n/a |
| Bracketing std. #1 ret. time rec. (%) | 100.06 | 100.01 | 100.06 | 100.05 | 100.03 | 100.05 | 100.03 | n/a |
| Bracketing std. #2 rel. dev. (%) | 4.51 | 1.18 | 4.01 | 1.12 | 3.80 | 3.78 | 3.86 | n/a |
| Bracketing std. #2 ret. time rec. (%) | 100.06 | 99.89 | 99.75 | 99.77 | 99.78 | 99.78 | 99.81 | n/a |

| Lab 9 - sequence #2 | DP1 | DP2 | DP3 | DP4 | DP5 | DP6 | DP7 | DP1-7 |
| --- | --- | --- | --- | --- | --- | --- | --- | --- |
| R^2^ | 1.00 | 1.00 | 1.00 | 1.00 | 1.00 | 1.00 | 0.99 | n/a |
| System suit. sample area (%RSD) | 0.66 | 1.66 | 0.96 | 2.67 | 1.51 | 3.20 | 4.13 | n/a |
| System suit. sample ret. time (%RSD) | 0.23 | 0.10 | 0.12 | 0.11 | 0.11 | 0.10 | 0.10 | n/a |
| Recovery (%) | n/a | n/a | n/a | n/a | n/a | n/a | n/a | 100.4 |
| Bracketing std. #1 area. rel. dev. (%) | 1.02 | 2.09 | 1.31 | 4.37 | 2.49 | 6.14 | 4.25 | n/a |
| Bracketing std. #1 ret. time rec. (%) | 100.08 | 100.10 | 100.02 | 100.02 | 100.06 | 100.07 | 100.08 | n/a |
| Bracketing std. #2 rel. dev. (%) | 1.65 | 1.37 | 0.42 | 2.53 | 0.82 | 1.94 | 14.75 | n/a |
| Bracketing std. #2 ret. time rec. (%) | 100.08 | 100.10 | 100.02 | 100.02 | 100.06 | 100.07 | 100.08 | n/a |

| Lab 10 | DP1 | DP2 | DP3 | DP4 | DP5 | DP6 | DP7 | DP1-7 |
| --- | --- | --- | --- | --- | --- | --- | --- | --- |
| R^2^ | 1.00 | 1.00 | 1.00 | 1.00 | 0.99 | 0.99 | 1.00 |  |
| System suit. sample area (%RSD) | 0.24 | 0.56 | 0.99 | 1.03 | 2.03 | 1.59 | 6.21 |  |
| System suit. sample ret. time (%RSD) | 0.15 | 0.09 | 0.04 | 0.06 | 0.09 | 0.11 | 0.07 |  |
| Recovery (%) |  |  |  |  |  |  |  | 95.5 |
| Bracketing std. #1 area. rel. dev. (%) | 1.09 | 3.60 | 4.72 | 11.65 | 14.70 | 19.69 | 3.63 |  |
| Bracketing std. #1 ret. time rec. (%) | 99.74 | 100.05 | 100.11 | 100.12 | 100.10 | 100.06 | 100.06 |  |
| Bracketing std. #2 rel. dev. (%) | 2.92 | 1.03 | 1.32 | 6.41 | 9.35 | 14.36 | 8.23 |  |
| Bracketing std. #2 ret. time rec. (%) | 99.64 | 99.99 | 100.02 | 99.98 | 100.02 | 100.08 | 100.15 |  |

End user feedback

Users submitted feedback alongside study results. Edits were made in the method to reflect these feedbacks including:

- Correct typos in method
- Specify what sample matrix require defatting
- Underline the importance that all solid must be wet with solvent before sonication
- Provide context for the detection condition optimization and the importance of system suitability
- Add warning to prevent SPE outlet to dip in solution, 15 mL get filled with 14.5 mL approximately
- Provide example chromatograms to show PMT gain optimization and peak integration details

Technical challenges while implementing the method were also reported. One laboratory observed significant baseline drift within each run. This was resolved by using a higher grade of acetic acid for the mobile phase. One laboratory failed system suitability during its first run, and this was associated with an inadequate refill of mobile phase during the run. This was resolved after sharing technical resources on the importance of system equilibration. Indeed, the AOAC 2020.05 method relies on HILIC for the separation of CF by degree of polymerization. This mode of separation is very sensitive to mobile phase composition changes which exclude the practice to refill a solvent bottle mid run and requires extensive system equilibration before any new experiment.
